# Understanding and Overcoming Osteosarcoma Heterogeneity

**DOI:** 10.3390/biom16060874

**Published:** 2026-06-15

**Authors:** Sukjoo Cho, Katherine Shelmidine, Jason T. Yustein

**Affiliations:** 1 Aflac Cancer and Blood Disorders Center, Children’s Healthcare of Atlanta, Atlanta, GA 30329, USA; 2 Department of Pediatrics, Emory University School of Medicine, Atlanta, GA 30322, USA; katherine.shelmidine@emory.edu; 3 Cancer Biology Program, Graduate Division of Biological and Biomedical Sciences, Emory University Laney Graduate School, Atlanta, GA 30322, USA

**Keywords:** osteosarcoma, sarcoma, heterogeneity, genomics, tumor microenvironment, lineage plasticity, biomarker, combinational therapy, maintenance therapy

## Abstract

Osteosarcoma (OS) is the most common primary bone cancer in adolescents and young adults. Despite tremendous preclinical and clinical efforts to advance therapy for OS, the standard of care, consisting of surgical resection and pre- and postoperative chemotherapy, has remained unchanged for over 40 years. Growing molecular understanding of OS highlights tumor heterogeneity as a major obstacle to therapeutic advances. In this narrative review, we comprehensively discuss current evidence of OS heterogeneity and strategies to overcome the barrier. Evidence shows that OS heterogeneity is multifactorial: it retains complex and dynamic somatic genomics, including genomic instability, alterations in tumor suppressors, and amplification/overexpression of oncogenes such as *MYC*. The tumor is associated with various germline vulnerabilities. OS’s tumor microenvironment has intense cellular and spatial diversity, which significantly shapes its heterogeneity. The effects of lineage plasticity, as well as epigenetic and metabolomic mechanisms, on OS heterogeneity are under study. To overcome this extreme heterogeneity, the therapeutic strategies for OS must be comprehensive and diversified. While surgical resection remains a mainstay of treatment, efforts to identify actionable biomarkers that guide risk stratification and therapy are ongoing. Diverse preclinical models offer insights into OS biology and novel therapeutics. To enhance combinational therapy for OS, various agents, including multi-targeted receptor tyrosine kinase inhibitors, immunotherapies, and epigenetic and metabolic modifiers, are being investigated. Distinctive efforts are continuing to establish maintenance therapy for OS. In summary, elucidating the complex drivers of OS heterogeneity, together with the development of multifaceted strategies to address them, is critical to accelerating therapeutic progress in OS.

## 1. Introduction

Osteosarcoma (OS) is an aggressive bone tumor that predominantly affects adolescents and young adults [1]. It is the most common primary bone cancer, albeit rare, with an estimated 400 new pediatric cases in the U.S. annually. The current standard treatment for OS, consisting of surgery and pre- and postoperative chemotherapy with methotrexate, doxorubicin, and cisplatin (MAP), was established in the 1980s [2,3]. The introduction of systemic chemotherapy increased the long-term survival of patients with localized disease to 70%. However, it did not significantly benefit those presenting with metastatic or relapsed disease, whose long-term survival remained less than 30% [1,4,5]. Accordingly, multiple large-cohort clinical trials investigated the addition of agents to MAP, including mifamurtide, pegylated interferon α-2b (IFN-α-2b), ifosfamide, and etoposide, unfortunately without significant positive outcomes [6,7,8]. Consequently, the standard of care for OS has remained largely unchanged for over 40 years.

**Figure 1:**
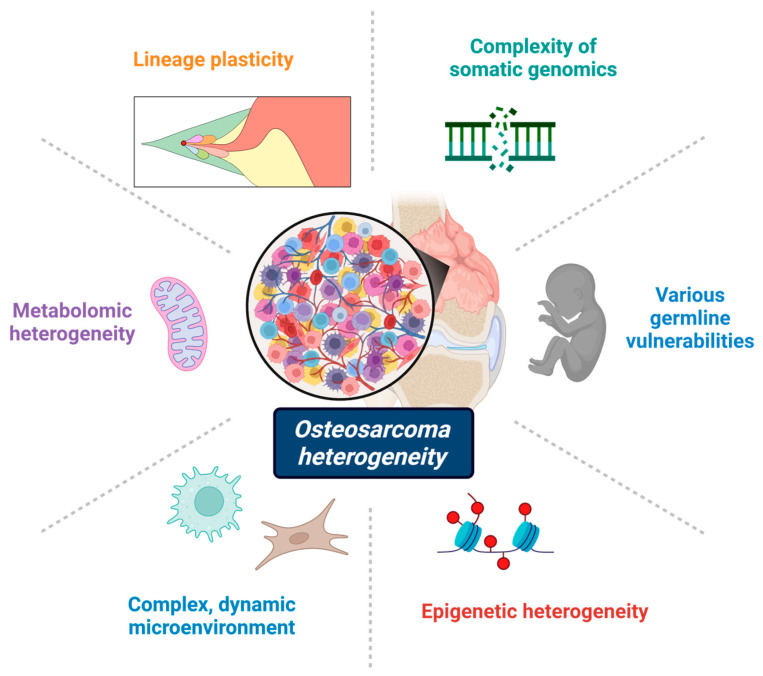
Various determinants of OS heterogeneity. Created with BioRender.com (https://www.biorender.com/, accessed on 4 June 2026).

Expanding molecular evidence of OS highlights tumor heterogeneity as a key determinant of such stagnant progress. Pan-cancer analyses demonstrated that OS has the most complex genomics among pediatric malignancies [9,10]. Extensive data described altered tumor suppressor genes, amplified oncogenes, and genomic instability of OS, together contributing to genomic complexity [11,12,13,14,15]. Furthermore, longitudinal analyses of patient samples demonstrated plastic tumor clonality that overcomes treatment and eventually leads to relapse [16]. At the cellular level, the role of cancer stem cells in OS heterogeneity and plasticity has been highlighted, which contributes to tumor growth, metastasis, and drug resistance [17]. Moreover, single-cell transcriptomic data revealed a cellularly and spatially distinct tumor microenvironment (TME) within individual tumors and across patients, overall immunosuppressive and treatment-resistant [18,19]. Collectively, OS appears to be extremely heterogeneous and plastic, making the current “one-size-fits-all” approach suboptimal. In this narrative review, we meticulously summarize current evidence of OS heterogeneity from genomic, epigenetic, TME, metabolomic, and clonality standpoints (Figure 1). Furthermore, discussions about multifaceted strategies to overcome such heterogeneity will follow.

## 2. Understanding OS Heterogeneity

### 2.1. Complexity of Somatic Genomics

OS displays one of the most complex genomic landscapes among childhood cancers, characterized by widespread copy-number alterations, structural rearrangements, and chromosomal instability, collectively leading to marked intra- and intertumoral heterogeneity. Loss or dysfunction of tumor suppressors disrupts critical processes such as cell cycle regulation, DNA damage response, chromatin remodeling, and oncogenic signaling pathways, thereby promoting genomic instability and enabling clonal evolution [11,12,14,15,16,20]. Copy-number alterations mostly involve tumor suppressor genes, including *TP53*, *RB1*, *ATRX*, and *PTEN*, which are difficult to target therapeutically. Whole-genome sequencing (WGS) studies showed that disrupted tumor suppressors often contribute to genomic instability in OS, leading to extensive structural alterations, including chromothripsis (chromosome shattering), kataegis (localized strand-specific hypermutation), and breakage-fusion-bridge cycles (fusion of broken ends from different chromatids or chromosomes) [11,21,22,23]. These catastrophic genomic events contribute to genetic diversity within the tumor, providing a substrate for the selection of aggressive and therapy-resistant clones [16,22].

Despite extensive structural genomic alterations, OS exhibits a relatively low frequency of recurrent single-nucleotide mutations compared to many adult carcinomas, resulting in intermediate tumor mutational burden (TMB) [9,11,24]. This creates a paradox that genomic complexity does not necessarily translate into robust immunogenicity. This highlights the importance of additional factors, such as neoantigen quality, immune infiltration, and TME composition, in determining therapeutic response [18,25].

Furthermore, unlike other malignancies, OS does not exhibit high-frequency oncogene-activating mutations that are often druggable. Instead, OS frequently exhibit amplification, which results in overexpression of oncogenes such as *PIK3CA*, *MTOR*, *AKT1* (PI3K-AKT–mTOR pathway), *IGF1R* (IGF pathway), *VEGFA* and *KDR* (VEGF pathway), *PDGFRA* (PDGF pathway), *KIT* (c-KIT pathway), *MYC* (c-MYC pathway), and *CDK4*, *CCNE1*, and *CCND2* (cell-cycle pathway) [24,26,27,28,29]. Some of these genes were observed in highly focal amplification (*IGF1R*, *CCNE1*, and *MYC*), while others were in larger segments of copy-number gain [30]. Such complex amplification patterns involving various oncogenes make therapeutic targeting extremely challenging.

#### MYC Drives Therapeutic Challenge in OS

Among the oncogenic drivers of OS, *c-MYC* amplification and/or overexpression, carried by approximately 20% of patients with OS, stands as a central regulator of aggressive tumor expansion and therapy resistance, ultimately determining prognosis [31,32]. *MYC* promotes cell proliferation by upregulating cyclin-dependent kinases (CDKs) and repressing cell cycle brakes [33,34]. This oncogene drives metabolic reprogramming by increasing oxidative phosphorylation, which supports tumor growth under metabolic stress [35]. In addition, *MYC* drives metastatic spread of OS by promoting migratory and invasive phenotypes, angiogenesis, and colonization at distant sites [36,37]. *MYC* also modulates the TME by influencing immune cell composition, including suppression of cytotoxic T cell activity and promotion of pro-tumorigenic macrophage phenotypes, further contributing to immune evasion [37,38]. Furthermore, in temporal WGS data of 37 samples from 11 patients with relapsed/refractory OS, *MYC* amplification was nominal at diagnosis, but emerged during therapy and dominated at relapse [16]. This observation underscores the role of *MYC* signatures in disease progression and treatment resistance via therapy-driven clonal selection. As such, amplification and/or overexpression of *MYC* are under active investigation as a biomarker for risk stratification [15,26].

Unfortunately, direct pharmacologic targeting of MYC remains challenging because it is a nuclear transcription factor that (1) is poorly accessible to larger therapeutics such as monoclonal antibodies, (2) has an intrinsically disordered structure lacking stable binding pockets for small molecules, and (3) lacks enzymatic activity, precluding inhibition by competitive catalytic drugs [39,40]. *MYC* is essential for normal cellular function, raising concerns about systemic toxicity with direct inhibition strategies. Nonetheless, efforts to target MYC-driven OS are ongoing. For instance, OMO-103, which directly interferes with MYC binding to DNA, is currently undergoing a phase II trial for advanced OS (ClinicalTrials.gov, NCT06650514).

Strategies to indirectly target *MYC*-driven OS are under active investigation. Targeting CDKs regulating cell cycle and oncogenic transcription has gained considerable interest [41]. While CDK4/6 inhibitors have acquired the most attention, preclinical data show that they lead to tumor growth inhibition, rather than regression [42]. Unsurprisingly, a phase II study that tested CDK4/6 inhibitor monotherapy (palbociclib) in relapsed/refractory pediatric solid tumors demonstrated no objective response [43]. Accordingly, efforts have been made to combine with additional targeted therapies, such as PI3K-mTOR inhibitors, which generated intriguing preclinical data [44,45]. Expanding targets to other CDK subgroups, such as CDK9, CDK12, and CDK13, also warrants further investigation [42]. Another potentially promising target is SUMOylation, a post-translational modification that regulates MYC [46,47,48]. Subasumstat (TAK-981), a first-in-class small molecule inhibiting SUMOylation, has shown efficacy in preclinical OS models [49,50].

### 2.2. Complexity of Germline Genomics

While most cases of OS are sporadic, a subset occur in individuals with established pathogenic germline variants (Table 1). Although such variants are not major drivers of OS, understanding their biological mechanisms provides additional insights into OS heterogeneity.

The best-known OS predisposition syndromes are Li–Fraumeni syndrome and hereditary retinoblastoma. These autosomal dominant disorders are caused by germline pathogenic variants in the tumor suppressor genes, *TP53* and *RB1*, respectively. Diamond–Blackfan anemia is another autosomal dominant disorder caused by variants in genes related to ribosomal RNA maturation. The exact molecular mechanisms by which haploinsufficiency of ribosomal protein genes promotes oncogenesis remain unclear. However, multiple downstream effects appear to contribute, including dysregulation of the p53 pathway and oncogenes, as well as oxidative stress resulting in DNA damage [62,63,64].

The autosomal recessive disorders with predisposition to OS harbor loss of function in DNA helicases, which are essential in repairing DNA and thus preserving genomic stability. Well recognized are *RECQL4* variants and their associations with Rothmund–Thomson and RAPADILINO syndromes [65]. *WRN* and *BLM* genes, whose variants result in Werner and Bloom syndromes, respectively, produce DNA helicases belonging to RECQ helicase subfamilies [66,67].

While these OS predisposition syndromes are extremely rare and highly penetrant, targeted and WGS/whole-exome sequencing (WES) studies revealed that variants in predisposition genes are more common in sporadic OS than expected. A *TP53*-targeted germline sequencing from unselected patients with OS showed that 10% of the patients diagnosed before 30 years of age carried germline *TP53* variants, which was higher than the previously reported 3–7% [68,69,70]. A landmark pan-cancer analysis investigating the landscape of predisposition genes in children observed 17.9% of OS has various germline mutations, with *TP53* variants being the most common [71]. A subsequent targeted sequencing/WES of 1244 patients with OS identified that 28% of the cohort carried such germline mutations [72].

In addition to the established germline variants, efforts to identify novel OS predisposing factors have been made. Particularly, *SMARCAL1*, which encodes a distinct DNA helicase, has drawn attention as a novel sarcoma predisposition gene in recent WGS/WES studies [73,74,75]. Furthermore, WES data of 2119 patients with OS demonstrated a 33-fold increased risk of OS development in individuals with germline *SMARCAL1* pathogenic variants [76]. Intriguingly, the study revealed improved overall survival in patients with *SMARCAL1* variants compared to those without the variants (hazard ratio (HR) 0.36 [95% confidence interval (CI) 0.14–0.96], *p* = 0.034). Future studies elucidating the mechanistic role of *SMARCAL1* in OS behavior will be insightful.

### 2.3. Epigenetic Heterogeneity

Epigenetic regulation contributes to OS heterogeneity by allowing gene expression levels to shift without impacting DNA sequence. Alterations in DNA methylation patterns, histone modifications, and chromatin remodeling are defining characteristics of individual OS subtypes with varying biological behavior [77]. Mutations in *ATRX* result in changes in chromatin remodeling proteins and add to epigenetic differences, while alterations in histone marks, such as H3K27me3 and H3K9me3, impact the modulation of gene expression [24,78,79,80,81,82]. These epigenetic alterations provide tumor cells with further flexibility to adapt to external environmental stressors and therapeutic pressure. Such plasticity enables dynamic switching between cellular states, contributing to intratumoral heterogeneity and complicating therapeutic targeting.

### 2.4. Heterogeneous TME

The OS TME is a complex and highly dynamic environment that consists of bone, stromal, immune, and vascular cells, together with mineralized extracellular matrix (ECM) [83,84]. Decades ago, studies leveraging electron microscopy revealed heterogeneous cellular profiles across OS tumors [85,86]. Recent single-cell RNA-sequencing (scRNA-seq) studies further demonstrated intratumoral heterogeneity by identifying multiple distinctive cell types, based on their gene expression profiles and canonical markers: osteoblasts, chondroblasts, osteoclasts, tumor-infiltrating lymphocytes (TILs), myeloid cells, cancer-associated fibroblasts (CAFs), mesenchymal stem cells (MSCs), adipocytes, and endothelial cells [18,87,88,89,90,91]. Of note, these studies highlighted intertumoral heterogeneity by distinguishing differences in cellular composition across the samples. Such extreme tumor heterogeneity of OS has been reiterated in studies exploiting spatial profiling [19,92,93,94,95].

OS retains an immunosuppressive TME shaped by low levels of TILs and enrichment of myeloid populations, particularly M2-macrophages and myeloid-derived suppressor cells (MDSCs) [18,25]. Recently, transcriptomic spatial profiling of nine pulmonary metastases demonstrated a spatially restricted inflammatory program enriched at the tumor periphery and diminished within the tumor [19]. In addition, the study observed abundant phagocytic tumor-associated macrophages (TAMs) in the peritumoral space, while the cells were absent intratumorally. Notably, immunosuppressive M2-macrophages are present both intra- and extratumorally. This distinct immune architecture highlights spatial heterogeneity contributing to the immunosuppressive nature of the disease.

Growing recognition of the TME has prompted efforts to integrate immune microenvironmental features into the assessment of therapeutic response and risk stratification. A study analyzing gene expression profiles demonstrated that higher expression of immune checkpoints, PDCD1LG2 (PD-L2) and HAVCR2 (TIM-3), together with increased infiltration of CD8+ cytotoxic T-cells, was associated with improved prognosis [96]. Moreover, a study leveraging deep learning-based digital pathology analysis with multiplex fluorescence immunohistochemistry (IHC) exhibited that spatial proximity between PD-1+ T-cells and PD-L1+ cells, rather than their overall abundance, determined response to immunotherapy [93]. A French study using conventional and multiplex IHC of 18 surgically removed OS specimens showed that the ratio of CD163+ histiocytes to CD68+ osteoclasts, osteoclast morphology, and CD8+ T-cell density were associated with response to neoadjuvant chemotherapy [92].

Interest in non-immune components of the TME is also growing. A study of gene expression profiles revealed that the level of CAFs and/or fibrosis was positively associated with CD8+ T-cell infiltration and chemotherapy response [96]. Recently, a study leveraging single-cell spatial transcriptomics and multiparameter immunofluorescence showed that lung metastasis induces fibrosis likely due to pathogenic, profibrotic epithelial intermediates and macrophages [94]. This study also showed that depositions of ECM proteins, such as fibronectin, were markedly increased in metastatic lesions and potentially targetable with an antifibrotic tyrosine kinase inhibitor. Another study that exploited paired tissue specimens (central and peripheral regions of the tumor) demonstrated that CLU+ endothelial cells, abundant in the tumor periphery, suppressed the anti-tumor immune response and promoted metastasis [97].

### 2.5. Metabolomic Heterogeneity

OS heterogeneity is significantly influenced by metabolic reprogramming. Metabolic adaptations allow tumor cells to survive under changing microenvironmental conditions, such as hypoxia, lack of nutrients, and treatment-induced stress. Specifically in OS, evidence shows that dysregulated glucose metabolism and inositol-related pathways lead to inherent metabolic variability within tumors [88,98]. Multi-omics analysis revealed OS subtypes with specific metabolic profiles, such as greater dependence on oxidative phosphorylation, particularly in *MYC*-amplified tumors [77]. Accordingly, MYC-driven subclones become enriched during treatment, thereby promoting metabolic diversification [16]. This enrichment reflects a dynamic process in which metabolomic heterogeneity in OS evolves over time, contributing to therapeutic resistance.

Altered lipid metabolism has also been observed in OS and other tumor types, where it supports major cellular functions, including membrane biosynthesis, energy storage, and redox balance [88,99]. Such processes help tumor cells cope with environmental challenges and therapeutic stress. The *MYC* oncogene appears to modulate fatty acid metabolism and drive tumor expansion [100]. Changes in tumor suppressors, such as TP53, further contribute to metabolic reprogramming and confer greater tolerance to cellular stress [101].

Furthermore, the TME significantly shapes tumor metabolic behavior. Metabolic conditions such as hypoxia affect downstream metabolic pathways, including anaerobic glycolysis and pentose phosphate pathways, thus determining redox balance, immune cell function, and therapy response in OS [20,88]. Studies indicate that competition for key nutrients like glucose between tumor and immune cells can impair immune cell function and promote an immunosuppressive TME, linking metabolomic heterogeneity to therapy response [20,102].

Collectively, these findings endorse a model where metabolic reprogramming contributes to the functional diversity of OS. Such mechanisms reflect the interplay between genetic alterations and external environmental factors, both of which influence disease advancement and response to treatment.

### 2.6. Lineage Plasticity

Cancer lineage plasticity refers to the capacity of tumors to adopt phenotypes that enable initiation, progression, metastasis, and therapy resistance of cancer [103]. Broadly, the results of lineage plasticity can be categorized as de-differentiation (adoption of a more primitive phenotype) and trans-differentiation (conversion from one mature phenotype to another, which may or may not require an interim de-differentiation step) [104]. Lineage plasticity can occur secondary to both tumor intrinsic factors, such as genetic, epigenetic, and transcriptional components, and extrinsic factors, including the TME, hypoxia, nutritional deficiency, and selective pressure from therapy [103]. This hallmark of cancer can greatly contribute to tumor heterogeneity as it may cause multiple phenotypes intra- and intertumorally.

The plasticity of OS has been typically viewed as an adaptive response to selective pressure from chemotherapy, resulting in treatment failure. Although incomplete, the mechanistic understanding of OS plasticity has been expanding. At the cellular level, epithelial-to-mesenchymal transition (EMT) and the reverse process, mesenchymal-to-epithelial transition (MET), have been recognized for their roles in promoting OS plasticity. Unlike carcinomas (cancers of epithelial origin), where EMT is often induced after drug exposure and triggers changes in tumor behavior, sarcomas are innately of mesenchymal origin and thus speculated to retain a plastic phenotype, enabling tumor cells to switch between epithelial and mesenchymal differentiation [105,106]. Such a plastic state promotes cancer stem cell generation, metastasis, and therapy resistance [107,108,109,110]. Reports have identified that various signaling pathways (WNT, NOTCH, JAK/STAT, MAPK, and TGF-β), along with >100 proteins and non-coding RNAs, were involved in EMT/MET regulation, which could be novel therapeutic targets [111].

At the genomic level, two conflicting models have been proposed to understand lineage plasticity of OS: dynamic vs. static models. The dynamic model speculates that continuous genomic instability imposes clonal evolution. For example, recent WGS data demonstrated that ongoing chromothripsis drives tumor evolution and heterogeneity [22]. In contrast, distinct evidence supports the static model, where an early catastrophic event defines genomic complexity of the tumor and is followed by stable maintenance of the genome. A longitudinal whole-genome single-cell DNA-sequencing study demonstrated that most somatic copy-number aberrations were acquired early in the oncogenic process and maintained from diagnosis to metastasis/relapse [16,112]. Chemotherapy such as cisplatin significantly increases mutational burdens, but such mutations do not drive therapy resistance [16,113].

These discrepant genomic-level observations suggest that components outside of the genome, such as epigenetic regulation, are contributing to OS lineage plasticity. A scRNA-seq study demonstrated that enrichment of genes related to histone methylation and acetylation was associated with trans-differentiation of malignant chondroblastic cells into malignant osteoblastic cells [18]. Notably, while both cell types were found in the primary tumor, only osteoblastic cells were identified in the lung lesion, suggesting a role of trans-differentiation in metastasis. Another recent study using Assay for Transposase-Accessible Chromatin Using Sequencing (ATAC-seq, which evaluates chromatin accessibility) identified two epigenetically distinct cell subtypes (early osteoblast-derived vs. late osteoblast-derived states) that coexist intratumorally, and show differential responses to targeted therapies [114]. For example, the study showed that the late osteoblast-derived subtype was responsive to a MEK inhibitor (trametinib), whereas the early osteoblast-derived subtype was more sensitive to an AURKB inhibitor (AZD1152).

The TME is another key stakeholder in OS lineage plasticity [115]. Particularly, the role of MSCs in OS stemness has been extensively studied, with the long-standing hypothesis that MSCs are the cellular origin of OS [116]. Evidence shows that the crosstalk between tumor cells and MSCs via various cytokines, including TGF-β1 and IL-6, as well as transcription factors such as NF-κB1, promotes tumor stemness [117,118,119,120]. A scRNA-seq analysis also highlighted the regulatory role of CAF on EMT via lysyl oxidase [121]. The plasticity of macrophages, the most abundant immune cells in the OS TME, has been well characterized [84]. Furthermore, a single-cell transcriptomic analysis demonstrated that tumor stemness is maintained by the communication between TAMs and OS stem cells through the IGF1-*RARRES2* axis [122].

## 3. Overcoming OS Heterogeneity

**Figure 2:**
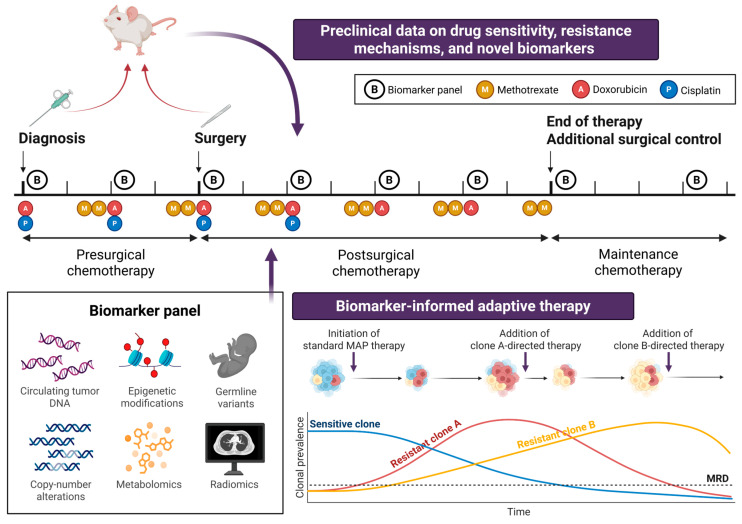
Therapeutic strategies to overcome OS heterogeneity. Various biomarkers collected via liquid biopsy and radiographic studies effectively guide adaptive therapy throughout the treatment course. Tissue samples obtained at diagnosis and surgery are leveraged to generate preclinical data on drug sensitivity, resistance mechanisms, and novel biomarkers, offering additional therapeutic avenues. Maintenance therapy follows the intensive therapy with MAP and adjunct agents to consolidate remission. Biomarkers are also used for MRD monitoring during off-therapy surveillance. Created with BioRender.com (https://www.biorender.com/, accessed on 4 June 2026).

As discussed so far, OS retains striking heterogeneity that complicates therapeutic targeting while promoting cancer growth and spread. In the remainder of this review, we present efforts to overcome such heterogeneity and future directions (Figure 2).

### 3.1. Surgical Resection

Surgery is the cornerstone of OS treatment. This statement remains valid even with the evolving molecular comprehension of this heterogeneous tumor. Intuitively, elimination of tumor bulk leads to instantaneous overthrow of tumor heterogeneity. This perspective is partly supported by long-standing evidence that complete removal of all evident disease yields the best long-term survival in patients with metastatic OS [123,124]. Moreover, retrospective studies indicate that repeat metastasectomy—even on multiple occasions when needed—plays a key role in extending the survival of patients with pulmonary relapse [125,126,127,128]. Given the significant inter- and intratumoral diversity among lung OS diseases, such observations highlight the critical merit of surgical control to subdue the disease heterogeneity [16].

Efforts to refine surgical management for OS are underway, particularly in the treatment of pulmonary metastases. The Children’s Oncology Group (COG) is conducting a phase III trial comparing open surgery (thoracotomy or sternotomy, the current mainstay) and minimally invasive surgery by thoracoscopy (NCT05235165). Additional interest has been given to fluorescence-guided surgery, which may help discern smaller tumors that appear indeterminate on scans, and save healthy lung tissues to the maximum extent [129,130].

Concerning the primary tumor resection, efforts have been made in limb salvage and reconstruction options, as well as implants and grafts [131,132]. Rotationplasty has acquired particular attention due to its superior postoperative function [133,134]. There has been progress in intraoperative guidance with fluorescence, radioluminescence, and computer-assisted navigation, as well as implants (e.g., 3D printing with novel materials, antimicrobial coating) [131]. While incorporating these innovations could be beneficial, little is known about their effects on the OS TME and long-term patient outcomes [135]. Thus, additional investigation is warranted before further incorporation into clinical practice.

### 3.2. Biomarker-Informed Adaptive Therapy

Combinational therapy targeting multiple different mechanisms of cancer is essential to overcome OS heterogeneity. Unfortunately, the current standard therapy with MAP yields futile outcomes in metastatic or recurrent/refractory OS. Multiple large-cohort clinical trials attempted to expand the cytotoxic combination by including ifosfamide and etoposide, with no measurable progress in survival (Table 2) [136,137].

Such prolonged stagnation in OS therapy is partly due to the absence of biomarkers that effectively guide risk-stratified therapy. Current non-molecular prognostic markers, such as metastatic status at diagnosis and histologic response to presurgical MAP, provide a vague sense of prognosis but do not direct subsequent management. Furthermore, histologic response to neoadjuvant MAP cannot be assessed until after approximately 12 weeks of treatment, thereby delaying timely risk assessment and treatment augmentation. As discussed earlier, *MYC* amplification and/or overexpression are actively being investigated for a potential prognostic role [29,32,142,143]. With difficulty in directly targeting MYC, however, therapy for MYC-driven OS remains unsettled.

In addition to the efforts to discover clinically actionable biomarkers, interest in biomarker-informed adaptive therapy has been growing, where therapeutic choice is repeatedly modified based on real-time patient-specific data (Figure 2) [144]. Through integration of comprehensive molecular/radiographic profiles, followed by tailored and/or augmented therapies, this approach can be the premier strategy to ultimately overcome OS heterogeneity. Unlike “one-size-fits-all” treatment, recognizing each tumor’s distinct therapeutic vulnerabilities and offering personalized therapy will help eradicate OS.

A key piece to concretize this strategy is effectively capturing measurable residual disease (MRD) that may lead to relapse. In pediatric leukemia, advances in MRD detection technologies, ranging from flow cytometry to next-generation sequencing (NGS), have been fundamental to the resounding success [145,146]. Unfortunately, optimal MRD detection methods for OS remain elusive. Currently, clinicians rely heavily on radiographic evaluation with magnetic resonance imaging (MRI) and computed tomography (CT). Unlike metaiodobenzylguanidine (MIBG) scan for neuroblastoma, however, these scans are not OS-specific and are less informative. Fibroblast activation protein inhibitor (FAPI) positron emission tomography (PET) has recently gained interest for its diagnostic and therapeutic potential for sarcomas [147,148,149]. However, OS-specific evidence remains scarce. Furthermore, the Response Evaluation Criteria in Solid Tumors (RECIST) criteria, widely accepted for disease evaluation in solid tumors, are not completely applicable to OS due to calcified osteoid matrix formation, which leads to a lack of measurable shrinkage [150,151]. Histologic evaluation with core or excisional biopsy is instructive, but occurs only at limited timepoints (diagnosis, resection, and relapse). Given that the target region of core biopsy is arbitrarily chosen, there is a chance that the molecular geography of the tumor is skewed.

Liquid biopsy to monitor circulating analytes in peripheral blood is an attractive modality that holds potential for effective MRD measurement in OS. A significant strength of the minimally invasive approach is real-time analysis of disease burden and therapeutic response that can promptly aid clinical decision-making [152]. While liquid biopsy can pick up various analytes, the current OS-specific evidence centers around circulating tumor DNA (ctDNA). Multiple studies have shown that the presence of pretreatment ctDNA is associated with inferior survival, particularly in localized OS [153,154,155]. COG is validating the prognostic role of ctDNA by assessing changes over time in an ongoing large-cohort prospective trial [156]. Given various emerging sequencing techniques, future studies are warranted to explore additional analytes (e.g., extracellular vesicles), coupled with multi-omics analysis [157]. Targeted approaches, such as polymerase chain reaction (PCR) to detect MYC copy, might also be intriguing [158,159,160].

Efforts to leverage artificial intelligence (AI) methods for precision oncology in OS are growing. Researchers have developed MRI-based radiomics models to characterize OS heterogeneity and predict survival, recurrence, and therapy response, generally using retrospective, small-cohort data [161,162,163,164,165,166,167,168]. One notable work was a pilot umbrella trial for refractory, metastatic OS in China, which directly translated AI-driven molecular subtyping into therapeutic exploration [77,169]. Although the outcome of this trial was modest, such application of AI to integrate genomic, epigenetic, and transcriptomic data for precision oncology is remarkable. AI models to incorporate multiple different data types into unified prediction systems warrant further validation in OS [170]. Combining AI with other novel technologies, such as blockchain and edge computing, may offer additional insights [171,172,173].

### 3.3. Preclinical Modeling

Modeling OS at the preclinical level is important to investigate hypothesis-driven molecular characterization of disease behavior and therapy resistance. In addition, preclinical models provide fundamental insights into the identification of biomarkers and novel therapeutics. However, due to the extensive tumor heterogeneity, accurate modeling of OS poses significant challenges. This heterogeneity spans genomic, cellular, and microenvironmental levels, making it difficult for any single model to fully capture the complexity of disease behavior and treatment response. While a broad range of preclinical OS models exists, including established cell lines, patient-derived cell lines, patient-derived xenografts (PDXs), and genetically engineered mouse models (GEMMs), there is no single model that perfectly represents all aspects of OS biology [174,175,176,177]. Therefore, it is critical to comprehensively integrate various model systems to capture specific elements of OS.

**Figure 3:**
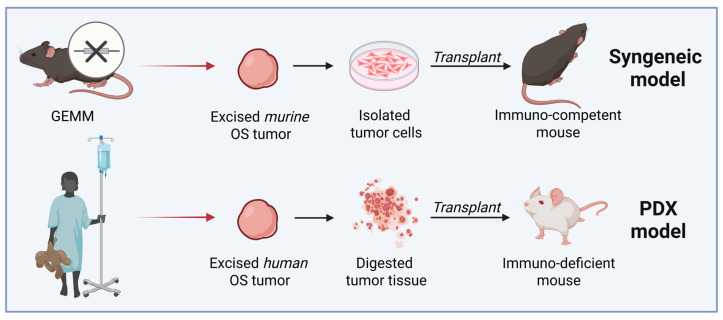
Comparison of GEMM-derived syngeneic model and PDX model. Created with BioRender.com (https://www.biorender.com/, accessed on 4 June 2026).

Because in vitro methods, such as 2D cell culture and 3D spheroids and organoids, are often limited in recapitulating the complexity of tumor architecture, including the TME, co-culture systems incorporating stromal or immune components enhance the investigation of tumor interactions with their microenvironment [178]. In contrast, in vivo methods offer a more accurate understanding of the diversity within OS. PDXs retain many characteristics of the original tumor, including genomic and phenotypic variations, whereas GEMMs allow for exploration of tumor development and growth within the framework of defined genetic changes (e.g., osteoblast-specific *Tp53* alteration and *Myc* knockin) [37,176,179] (Figure 3). Orthotopic engraftment, often into the tibia of mice, more precisely mimics the bone microenvironment and metastatic behavior compared to heterotopic engraftment. When implanted into immunocompetent mice, notably, GEMMs can make syngeneic models, which serve as powerful resources for immune-based studies [88,94,180,181]. Furthermore, co-clinical trial platforms, characterized by parallel implementation of preclinical and clinical trials leveraging PDXs and/or GEMMs, may provide ample insights into the identification of biomarkers, resistance mechanisms, and novel therapeutics [182,183,184,185,186,187,188].

### 3.4. Enhancing Combinational Therapy

**Figure 4:**
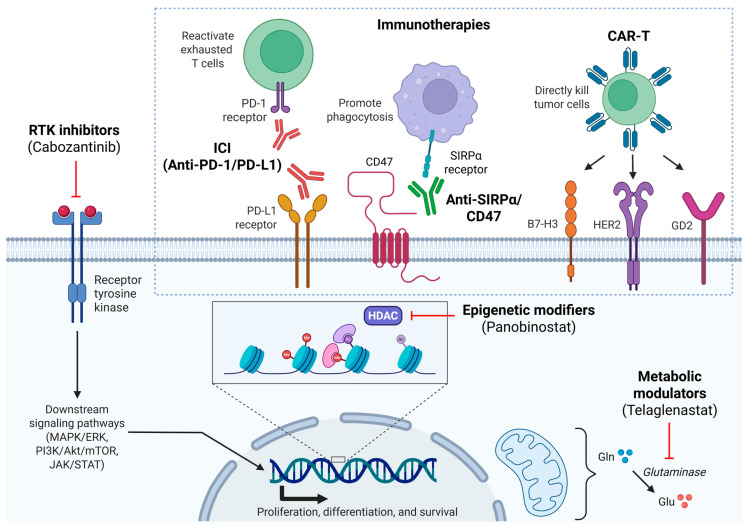
Emerging new agents for OS holding priority for clinical investigation. Created with BioRender.com (https://www.biorender.com/, accessed on 4 June 2026).

Combinational therapy integrating standard-of-care chemotherapy and novel targeted agents is essential for overcoming OS heterogeneity. Such multimodal approaches leverage synergistic effects to target diverse oncogenic mechanisms [189]. The limited efficacy of current therapies, particularly in relapsed and metastatic disease, underscores the need for strategies that address both tumor heterogeneity and adaptive resistance. In 2021, a report from the COG New Agents for OS Task Force identified several priority therapeutic modalities warranting further clinical investigation: multi-target tyrosine kinase (RTK) inhibitors, immunotherapies including macrophage checkpoint inhibitors, metabolic modulators, and epigenetic modifiers (Figure 4) [190]. Based on the improved understanding of OS heterogeneity, more recent studies have focused on rational combinational strategies including these agents [191,192]. Here, we revisit the drugs of interest from the COG report and discuss interim progress. Moreover, additional novel agents are further discussed.

#### 3.4.1. RTK Inhibitors

RTK inhibitors are often referred to as “multi-targeted” because many RTKs share common intracellular domains and thus a single inhibitor can simultaneously affect multiple downstream signaling pathways. These small molecules affect multiple signaling pathways that are critical to sarcomagenesis [193]. Among multiple RTK inhibitors tested in phase II trials for relapsed/refractory OS, sorafenib coupled with an mTOR inhibitor (everolimus), regorafenib, and cabozantinib demonstrated efficacy against OS [194,195,196,197,198]. Because of the toxicity profiles, COG selected cabozantinib and is conducting a phase II/III trial to investigate the upfront addition of the drug to standard MAP in patients with newly diagnosed OS (NCT05691478) [190].

More recently, additional interest has been given to combinational therapy with immune checkpoint inhibitors (ICIs), primarily to overcome immunotherapy resistance. In a single-arm trial investigating lenvatinib plus pembrolizumab for various sarcomas, for instance, a durable response was observed in a subset of patients with OS [199]. Specifically, this combination halted progression for >1 year in two of six trial participants with OS. A phase II trial testing cabozantinib in combination with atezolizumab in adolescents and young adults with advanced OS is also running (NCT05019703).

#### 3.4.2. Immunotherapies

Given the relatively high TMB of OS compared to that of other pediatric cancers, considerable attention has been given to therapeutic strategies incorporating ICI therapy [9,10]. Although multiple phase II trials evaluated ICI therapies in advanced OS, the results have shown an overall lack of efficacy [200,201,202,203]. This is likely secondary to a lack of neoantigen burden associated with high-level nonsense-mediated decay, along with immunosuppressive TME, overall mitigating anti-tumor immunity [25]. Therefore, augmentation of ICI therapy with additional treatment modalities, including radiation, CD73 inhibition, and multi-targeted RTK inhibitors, is being explored [204] (NCT04668300, NCT05019703).

Targeting immune checkpoints on the TAM is an emerging immunotherapy approach that unleashes macrophages to phagocytose tumor cells by inhibiting “don’t eat me” signals [205]. CD47 and CD24 expressed on tumor cells are the most representative such signals that respectively bind to signal regulatory protein α (SIRPα) and sialic acid-binding Ig-like lectin (Siglec-10), paired checkpoints on macrophages. Preclinical evidence demonstrated that monoclonal antibody therapy against CD47 enhances macrophage-mediated clearance of solid tumors, including OS [206,207]. Furthermore, double blockade of CD47 and GD2 showed synergistic effects, both in vitro and in vivo [208]. Unfortunately, a phase I trial testing such a combination with magrolimab (anti-CD47) and dinutuximab (anti-GD2) in relapsed OS was initiated but suspended early due to lack of efficacy and unacceptable toxicities (NCT04751383). Investigation for other macrophage checkpoint targeting mechanisms and additional combinational therapy is warranted.

Despite inconclusive survival benefit found in prior trials, mifamurtide remains attractive as a potential adjunct therapy. Derived from the cell wall of Bacille Calmette–Guerin, mifamurtide exhibited tumoricidal activity by stimulating macrophages in murine and canine models [209,210,211]. Given the preclinical evidence, the drug was tested in combination with MAP with/without ifosfamide in a COG-led phase III trial for newly diagnosed OS. While the initial analysis of this trial reported no benefit of adding mifamurtide, a subsequent analysis with longer follow-up demonstrated that six-year overall survival was improved from 70% to 78% in localized OS [6,212]. The addition of mifamurtide did not improve survival in metastatic OS [213]. Such results were favored by the European Medicines of Agency, leading to approval of the drug. However, the U.S. Food and Drug Administration disagreed with this decision. Nevertheless, given the emerging recognition of macrophages in OS TME, mifamurtide may provide an intriguing chance for novel combinational therapy.

Another immunotherapy of interest for OS is cell therapy. With resounding successes in B-cell acute lymphoblastic leukemia and neuroblastoma, in particular, chimeric antigen receptor-expressing T cells (CAR-Ts) have gained substantial preclinical momentum [214,215,216]. However, investigation of CAR-T therapy for OS remains mostly at pre-clinical or very early clinical phases. The major barriers include antigen-related challenges (e.g., low antigen specificity, heterogeneity, and escape mechanisms), immunosuppressive TME, TME-associated T-cell trafficking challenges, exhaustion and poor durability of T-cells, and on- and off-target toxicities [217,218]. Efforts to identify optimal targets for OS have been continuing, while HER2, B7-H3 (CD276), and GD2 have acquired the most interest [219,220,221,222]. Furthermore, interest in understanding microenvironmental factors in support of CAR-T is growing. Several ongoing early-phase trials aim to test conditioning regimens (e.g., fludarabine/cyclophosphamide and/or radiation), refining CAR-T design (e.g., bispecific B7-H3/CD19 CAR-T, cytokine-armored GD2 CAR-T), and combining CAR-T with an ICI (NCT04897321, NCT07222735, NCT04483778, NCT03721068) [208]. Notably, a recent report demonstrated that CD70 in the setting of EMT signatures can be effectively targeted with a highly sensitive CAR-T design, making CD70-directed CAR-T an attractive novel approach for OS [223].

#### 3.4.3. Metabolic Modulators

Metabolic reprogramming is a key feature of OS biology, contributing to heterogeneity. Particularly, glutamine serves as a fuel in tumor growth and spread [224]. A multi-omics study identified distinct metabolic dependencies in the MYC-driven OS subtype, including increased reliance on oxidative phosphorylation [77]. Telaglenastat (CB-839), a glutaminase blocker that inhibits mitochondrial oxidative phosphorylation, demonstrated anti-tumor effects in preclinical models for multiple cancers, including OS [225,226]. The drug has been tested in combination with other agents for various cancers; however, no trial dedicated to OS has been initiated [190]. Given the emerging understanding of metabolic reprogramming in OS, targeting metabolic pathways in conjunction with other drug classes has the potential to show significant response in the subset of OS tumors [88,227,228].

#### 3.4.4. Epigenetic Modifiers

Epigenetic dysregulation contributes to OS progression and plasticity. Panobinostat, a histone deacetylase (HDAC) inhibitor, demonstrated anti-tumor activity in preclinical OS models, as monotherapy and adjunct therapy to cytotoxic agents [229,230,231,232]. Notably, its combination with epirubicin, a novel topoisomerase II inhibitor, was tested in a phase I trial for adult soft tissue sarcomas with limited benefits [233]. DNA methyltransferase inhibitors that lead to hypomethylation are another epigenetic modifier of interest due to preclinical evidence, warranting clinical investigation [234].

#### 3.4.5. Additional Agents

Alterations in DNA repair pathways, such as *RB1* and *BRCA*, have drawn attention as a potential therapeutic target in a subset of OS. Despite strong preclinical rationale for poly ADP-ribose polymerase (PARP) inhibitors in these tumors, phase I and II studies of olaparib monotherapy in advanced pediatric solid tumors did not demonstrate significant efficacy [15,235,236,237,238]. Nonetheless, combining a PARP inhibitor with other treatment modalities warrants further investigation. For example, an Italian phase I study that tested a combination of olaparib and a novel alkylating agent, trabectedin, in patients with advanced, non-resectable bone and soft tissue sarcomas demonstrated a partial response in 14% of the enrollees [239].

Strategies to improve drug delivery have acquired considerable interest. OS has unique therapeutic challenges due to its physical microenvironment, collectively shaped by rapid bone remodeling, stiff and dense ECM, and altered proteoglycan composition [83]. Therefore, novel drug delivery platforms, including various nanoparticles, stimuli-responsive drug release, injectable hydrogels, and scaffolds, are under study, largely in the preclinical stage [240].

### 3.5. Maintenance Therapy

Continuous dosing of low-intensity anti-cancer drug(s), or maintenance therapy, has been perceived as a compelling option to perpetuate remission and achieve a durable response in various cancers. It is well accepted in hematologic malignancies, particularly leukemias, and certain solid tumors [241,242,243,244,245]. Many maintenance therapies established in the modern era target specific mutations or signaling of the tumor: imatinib, blocking BCR-ABL1 tyrosine kinase that drives chronic myeloid leukemia, and ibrutinib, inhibiting Bruton’s tyrosine kinase, critical in chronic lymphoblastic leukemia pathogenesis. This targeted maintenance monotherapy may not be optimal to conquer a heterogeneous cancer like OS. Conversely, maintenance therapy for acute lymphoblastic leukemia, two years of antimetabolites combined with intermittent steroid pulses and vincristine following the intensive upfront therapy, was empirically derived in the 1970s without a complete understanding of the necessity, ideal duration, or most effective composition [241]. Nonetheless, implementing multimodal maintenance therapy was proven to be more efficacious than repeating high-intensity therapy [246].

Early efforts to establish maintenance therapy for OS were unfruitful. EURAMOS-1 tested maintenance IFN-α-2b to stimulate anti-tumor immunity after completion of standard MAP in good responders (≥90% necrosis) and demonstrated no survival benefit [7]. Notably, a considerable number of patients had to stop IFN-α-2b early due to toxicity. Despite such disappointing results, endeavors to identify optimal maintenance therapy for OS are continuing and are summarized below.

#### 3.5.1. RTK Inhibitor Monotherapy

The multi-targeting nature of RTK inhibitors is an attractive maintenance therapy option for the tumor, even as monotherapy. Given the activity demonstrated in phase II trials for heavily pretreated high-risk OS, two phase II trials are ongoing to evaluate regorafenib as maintenance treatment in distinctive settings [195,196]. REGOMAIN is to assess maintenance regorafenib in patients with residual disease after ≥1 line of therapy (NCT04698785). REGOSTA is to compare regorafenib vs. observation in those who achieved remission after the first-line treatment (NCT04055220). Cabozantinib is another RTK inhibitor being evaluated as maintenance therapy in the ongoing COG trial for newly diagnosed OS (NCT05691478). The study aims to evaluate six months of maintenance cabozantinib monotherapy after completing standard MAP with/without cabozantinib.

#### 3.5.2. Metronomic Chemotherapy

Another maintenance therapeutic option under investigation is metronomic chemotherapy. Whereas cancer treatment has traditionally centered on delivering cytotoxic drugs at or near the maximum tolerated doses, metronomic chemotherapy involves constant administration of low-dose cytotoxic agents, typically in combination with other anti-cancer drugs targeting different aspects of neoplasm, such as angiogenesis inhibitors [247]. Targeting multiple distinct mechanisms of malignancy may control or diminish the burden of heterogeneous tumors. Furthermore, the metronomic approach could avoid not only the toxicity of high-dose chemotherapy but also the rest periods that are inevitable in the conventional counterpart, which is overall ideal for a maintenance strategy.

Metronomic chemotherapy has been studied as an attractive treatment option in low- and middle-income countries, especially for patients with recurrent/refractory disease, with unfruitful results. An Indian trial evaluated daily celecoxib and thalidomide with alternating periods of oral etoposide and cyclophosphamide in children with extracranial solid tumors, including 72 bone sarcomas. Unfortunately, this regimen failed to demonstrate a progression-free survival benefit in patients with OS [248]. A Moroccan trial investigated oral cyclophosphamide/etoposide and valproate in children with various relapsed/refractory cancers and found no response in 14 OS patients [249].

Efforts in the United States echoed the limited activity of metronomic chemotherapy in advanced OS. Continuous sirolimus, an mTOR inhibitor, and celecoxib, in combination with alternating periods of oral etoposide and cyclophosphamide (CHOAnome), have been tested in children with recurrent/refractory solid and brain tumors [250]. Of note, the mTOR pathway was highlighted as a common vulnerability in OS [24]. In the phase II trial, the best overall response of four patients with OS was progressive disease, while individuals with other diagnoses achieved prolonged stable disease [251].

Additional efforts have been made to leverage metronomic chemotherapy to consolidate remission and prevent relapse. A Latin American trial tested the addition of 1.4-year-long oral methotrexate and cyclophosphamide following the standard MAP regimen in patients with nonmetastatic, operable OS of the extremities [252], but found no benefit in event-free survival. Such results need to be viewed with caution because the prognosis of localized, resectable disease is generally favorable. CHOAnome is being investigated in children with high-risk solid tumors (e.g., metastatic OS) in remission after completion of first- or second-line treatment (NCT04469530). The study aims to prolong progression-free survival at two years.

## 4. Challenges and Future Directions

The scarcity of the disease is a major challenge to studying OS heterogeneity, particularly in the context of clinical investigation. While molecular subgrouping is essential in the evaluation of any heterogeneous malignancy, it will underpower the clinical trial and decrease the chance of positive results [253]. Therefore, distinctive considerations have been given to innovative trial designs and various endpoints beyond the RECIST criteria [254,255]. The National Cancer Institute-COG Pediatric Molecular Analysis for Therapy Choice (MATCH) Trial was a landmark precision oncology protocol that exploited NGS to identify actionable genetic alterations in refractory childhood cancers and facilitated timely investigation of multiple targeted therapies [256]. When it comes to OS-specific results, unfortunately, no objective response was observed across the arms, implying a pressing need to test rational combinational therapy options [43,238,257,258,259]. Currently, the combination of cabozantinib and MAP is under investigation by COG (NCT05691478). Another study worth noting is the OstEvo (Osteosarcoma Evolves) trial testing the addition of four different targeted therapies (gefitinib, trametinib, disulfiram, and sunitinib) to MAP in high-risk disease (NCT07477457). The protocol also aims to discern lineage plasticity during therapy by leveraging multi-omics analyses, which could be extremely impactful.

The role of preclinical investigation is fundamental in the generation of critical knowledge to overcome OS heterogeneity, including actionable biomarkers, mechanisms of treatment resistance, and efficacious combinational therapy. However, a major challenge here is the discordance across various model systems and methodologies [260]. Of note, each preclinical model represents different aspects of OS biology and thus remains complementary. Coupled with data generated from patient samples, therefore, integration of multi-omics data of different model systems will offer deeper insights. Utilization of readily available techniques should be considered to facilitate this integration.

Our review also has limitations. As a narrative review, we acknowledge potential bias in the selection process of literature. Relevant studies might have been missed due to language or the search database. In addition, publication bias could have affected the existing evidence and resultant discussions in this paper.

## 5. Conclusions

In summary, OS is the most common bone cancer characterized by intense intra- and inter-tumoral heterogeneity, which contributes to aggressive tumor behavior and poor therapy response. Such heterogeneity is shaped by numerous tumor intrinsic and extrinsic components and determines treatment response and prognosis. To conquer this complexity, tremendous efforts have been made to improve surgical methods, establish effective and clinically actionable biomarkers, and expand therapeutic options. Coupled with comprehensive preclinical models and novel AI-driven technologies, scientific discoveries will be accelerated and lead to clinical impacts. Despite the intricacy of OS biology that has impeded therapeutic progress for decades, the optimism in the pursuit of disease control has been relentless. We believe rapidly expanding knowledge on OS heterogeneity has the potential to revolutionize the care of patients with OS and eventually defeat this aggressive disease.

## Figures and Tables

**Table 1:** Established OS predisposition syndromes.

Syndrome	Phenotype	Gene	Location	Function
Autosomal dominant disorders				
Li–Fraumeni syndrome (OMIM 151623) [51]	Early onset of tumors, multiple tumors within an individual, various tumor types, and multiple affected family members.	*TP53*	17p13.1	DNA damage repair
Hereditary retinoblastoma (OMIM 180200) [52,53,54]	Embryonic malignant neoplasm of retinal origin, mostly early childhood onset, often bilateral.	*RB1*	13q14.2	Cell-cycle checkpoint
Diamond–Blackfan anemia (OMIM 105650) [55,56]	Inherited red cell aplasia with typical onset during infancy. Often associated with congenital craniofacial and thumb malformations and short stature. Retains increased risk of multiple malignancies, including myelodysplastic syndrome.	Various pathogenic variants of the genes involved in ribosomal RNA maturation		
Autosomal recessive disorders				
Rothmund–Thomson syndrome, type 2 (OMIM 268400) [57]	Poikiloderma (distinguishing feature from RAPADILINO syndrome), congenital bone defects, and increased risk of OS in childhood and skin cancer later in life.	*RECQL4*	8q24.3	DNA helicase
RAPADILINO syndrome (OMIM 266280) [58]	RAdial ray defects, absent/hypoplastic PAtellas, cleft/highly arched PAlate, DIslocated joints, short stature (LIttle size), slender NOse and NOrmal intelligence. Absent poikiloderma.	*RECQL4*	8q24.3	DNA helicase
Werner syndrome (OMIM 277700) [59,60]	Appearance (hair change, atrophy of skin and subcutaneous fat) and disorders (cataracts, diabetes mellitus, osteoporosis, premature arteriosclerosis, and various cancers) of accelerated aging.	*WRN*	8p12-p11.2	DNA helicase/ exonuclease
Bloom syndrome (OMIM 210900) [61]	Pre- and postnatal growth deficiency; skin photosensitivity; immune deficiency; insulin resistance/increased risk for diabetes; and significantly increased risk of early onset of cancer and for the development of multiple cancers.	*BLM*	15q26.1	DNA helicase

**Table 2:** Key late-phase clinical trials that evaluated ifosfamide and etoposide for OS.

Trial (Year of Report)	Study Design (*n* of IE Recipients)	Chemotherapy (g/m^2^)		Outcome	Severe Therapy- Related Toxicity
		Presurgical	Postsurgical		
POG9450 (2002) [138]	An international phase II/III single-arm trial for patients aged <30 years with newly diagnosed metastatic OS (*n* = 43).	I: 35 E: 1	M: 120 A: 0.375 P: 0.48 I: 36 E: 1.5	CR: 10% PR: 49% ORR: 59% (±8%)	Neutropenia: 83% Thrombocytopenia: 29% Sepsis: 24% Death: 5%
SFOP-OS94 (2007) [139]	A French phase III randomized trial for patients aged <20 years with newly diagnosed non-metastatic OS comparing presurgical MA and MIE. Poor responders to pre-surgical MA and MIE (defined as >5% viable tumor) received IE and AP after surgery, respectively (*n* = 189).	[MA] M: 84 A: 0.14	[Good vs. poor response to MA] M: 144 vs. 0 A: 0.21 vs. 0 I: 0 vs. 70 E: 0 vs. 0.375	MIE led to better histologic response at surgical resection than MA (56% vs. 39%, *p* = 0.009). However, no significant intergroup difference in 5-year event-free and overall survival was observed.	Acute toxicity was overall manageable. No therapy-related death occurred.
		[MIE] M: 84 I: 28 E: 0.15	[Good vs. poor response to MIE] M: 144 vs. 0 A: 0 vs. 0.35 P: 0 vs. 0.6 I: 36 vs. 0 E: 0.225 vs. 0		
EURAMOS-1 (2016) [8]	An international phase III randomized trial, where patients aged ≤25 years with newly diagnosed OS with poor response to presurgical MAP (≥10% viable tumor) were treated with MAP vs. An international phase III randomized trial, where patients aged ≤25 years with newly diagnosed OS that showed poor response to presurgical MAP (≥10% viable tumor) were treated with MAP vs. MAPIE (*n* = 308).	M: 48 A: 0.15 P: 0.24	M: 96 A: 0.3 P: 0.24 with/without I: 60 E: 1.5	EFS did not differ between MAP and MAPIE groups (HR 0.98 [95% CI 0.78–1.23]).	Neutropenia: 90% Thrombocytopenia: 83% Febrile neutropenia without documented infection: 73% Death: 1%
OS2006 (2018) [140,141]	A French phase III randomized trial, where some patients aged ≤25 years were treated with presurgical MIE, followed by MIE vs. MAP postoperatively, depending on the risk of treatment failure (*n =* 409).	M: 84 I: 24 E: 0.6	[Good response (<10% viable tumor) in localized resected disease] M: 144 I: 36 E: 0.6	This regimen achieved 5-year event-free (56%) and overall survival (71%), comparable to those with MAP, while avoiding AP in 59% of the study participants.	Most patients had ≥1 severe acute toxicity, including neutropenia with/without fever, hepatotoxicity, and thrombocytopenia. In total, 5 patients died, 4 of whom received AP.
			[Poor response (≥10% viable tumor) or metastatic/unresectable disease] M: 60 A: 0.375 P: 0.6		

A, doxorubicin; CR, complete response; E, etoposide; I, ifosfamide; M, methotrexate; *n*, number; ORR, overall response rate; OS, osteosarcoma; P, cisplatin; PR, partial response.

## Data Availability

No new data were created or analyzed in this study. Data sharing is not applicable to this article.

## Abbreviations

The following abbreviations are used in this manuscript:

AI	Artificial intelligence
ATRX	Alpha-thalassemia/mental retardation syndrome, X-linked
AURKB	Aurora kinase B
B7-H3	B7 homolog 3
CAF	Cancer-associated fibroblast
CAR-T	Chimeric antigen receptor-expressing T cell
CD	Cluster of differentiation
CDK	Cyclin-dependent kinase
CI	Confidence interval
CLU	Clusterin
COG	Children’s Oncology Group
CT	Computed tomography
ctDNA	Circulating tumor DNA
CXCR3	CXC motif chemokine receptor 3
DNA	Deoxyribonucleic acid
ECM	Extracellular matrix
EMT	Epithelial-to-mesenchymal transition
FAPI	Fibroblast activation protein inhibitor
GEMM	Genetically engineered mouse model
GD2	Disialoganglioside
Gln	Glutamine
Glu	Glutamate
HAVCR2	Hepatitis A virus cellular receptor 2
HDAC	Histone deacetylase
HER2	Human epidermal growth factor receptor 2
HR	Hazard ratio
ICI	Immune checkpoint inhibitor
IFN-α-2b	Pegylated interferon α-2b
IGF	Insulin-like growth factor
IHC	Immunohistochemistry
IL	Interleukin
JAK	Janus kinase
MAP	Methotrexate, doxorubicin, and cisplatin
MAPK	Mitogen-activated protein kinase
MDSC	Myeloid-derived suppressor cell
MEK	Mitogen-activated protein kinase kinase
MET	Mesenchymal-to-epithelial transition
MIBG	Metaiodobenzylguanidine
MRI	Magnetic resonance imaging
MRD	Measurable residual disease
MSC	Mesenchymal stem cell
mTOR	Mammalian-target of rapamycin
NCI	National Cancer Institute
NF-κB1	Nuclear factor κ subunit 1
NGS	Next-generation sequencing
OS	Osteosarcoma
PCR	Polymerase chain reaction
PD-1	Programmed cell death protein 1
PD-L1	Programmed death-ligand 1
PD-L2	Programmed death-ligand 2
PDCD1LG2	Programmed cell death 1 ligand 2
PDGF	Platelet-derived growth factor
PDX	Patient-derived xenograft
PET	Positron emission tomography
PI3K	Phosphatidylinositol-3-kinase
RB1	Retinoblastoma 1
RECIST	Response Evaluation Criteria in Solid Tumors
RNA	Ribonucleic acid
RTK	Receptor tyrosine kinase
scRNA-seq	Single-cell RNA-sequencing
SMARC	SWI/SNF-related, matrix-associated, actin-dependent regulators of chromatin
STAT	Signal transducer and activator of transcription
TAM	Tumor-associated macrophage
TGF-β	Transforming growth factor-β
TIM-3	T-cell immunoglobulin and mucin-domain containing-3
TIL	Tumor-infiltrating lymphocyte
TMB	Tumor mutational burden
TME	Tumor microenvironment
TP53	Tumor protein p53
VEGF	Vascular endothelial growth factor
WES	Whole-exome sequencing
WGS	Whole-genome sequencing
